# The kin17 Protein in Murine Melanoma Cells

**DOI:** 10.3390/ijms161126072

**Published:** 2015-11-24

**Authors:** Anelise C. Ramos, Vanessa P. Gaspar, Sabrina M. G. Kelmer, Tarciso A. Sellani, Ana G. U. Batista, Quirino A. De Lima Neto, Elaine G. Rodrigues, Maria A. Fernandez

**Affiliations:** 1 Departamento de Biotecnologia, Genética e Biologia Celular, Universidade Estadual de Maringá, 87020-900 Maringá, Paraná, Brasil; anelise.andre@gmail.com (A.C.R.); vapigas@gmail.com (V.P.G.); sabrinamg12@hotmail.com (S.M.G.K.); qalneto@gmail.com (Q.A.D.L.N.); 2 Laboratório de Imunobiologia do Câncer, Departamento de Microbiologia, Imunologia e Parasitologia, Escola Paulista de Medicina, Universidade Federal de São Paulo, 04023-062 São Paulo, São Paulo, Brasil; tarcis.sellani@gmail.com (T.A.S.); anagurbanin@gmail.com (A.G.U.B.); rodrigues.elaine@unifesp.br (E.G.R.)

**Keywords:** kin17 protein, melanoma cells, DNA metabolism

## Abstract

kin17 has been described as a protein involved in the processes of DNA replication initiation, DNA recombination, and DNA repair. kin17 has been studied as a potential molecular marker of breast cancer. This work reports the detection and localization of this protein in the murine melanoma cell line B16F10-Nex2 and in two derived subclones with different metastatic potential, B16-8HR and B16-10CR. Nuclear and chromatin-associated protein fractions were analyzed, and kin17 was detected in all fractions, with an elevated concentration observed in the chromatin-associated fraction of the clone with low metastatic potential, suggesting that the kin17 expression level could be a marker of melanoma.

## 1. Introduction

The detection and treatment of melanoma can be considered one of the major challenges in oncology. The variability at the molecular level and the cellular heterogeneity of this disease is a major hindrance to tumor therapy [1]. Several studies have been carried out to understand this heterogeneity and attempt a classification into subtypes [2], which may lead to better treatment choices for individual patients.

Proteins associated with DNA repair are among the best-studied targets in malignant tumors [3]. The repair protein kin17 is overexpressed in breast cancer [4], as well as in several other human cell lines such as H1299 (lung cancer), RKO (colorectal carcinoma), K562 (chronic myeloid leukemia), and HEK 293 cells (kidney embryonic cells) [5]. Compared with 16 cell lines from different types of tumors, the human melanoma cell line MeWo, derived from a lymph node metastasis, exhibited the lowest expression of the kin17 protein; however, a primary human melanoma cell line was not included in this study [5].

The kin17 protein is described as being involved in the processes of DNA replication initiation, recombination, and repair in mammals [5,6,7,8]. A significant number of reports have suggested that kin17 is activated by stress caused by gamma or UVC irradiation [6,9]. This protein has been described in many organisms, with 92.4% homology between mice and humans, with 391 *vs.* 393 amino acids and a molecular mass of 45 *vs.* 47 kDa, respectively; these two proteins also exhibited the same isoelectric point of 9.3 [8,10,11,12,13]. Structural analysis described five functional domains: a nuclear localization signal; a zinc finger motif (Cx_2_Cx_12_Hx_5_H) that allows the protein to interact with DNA; a region of 49% homology with the C-terminal end of the bacterial RecA; and a KOW (Kyprides, Ouzounis, Woese) tail C-terminus, which is related to protein–protein and RNA–protein association [13,14,15,16].

Cloutier *et al.* [17,18] described a different function for the kin17 protein. These authors proposed that a methyltransferase is associated with this protein that can methylate lysine 137, regulating the transit of the kin17 protein between the cytoplasm and the nucleus. Kin17 has also been reported to exhibit chaperone activity or to interact with other chaperone proteins, altering their activity. Although the biochemistry and structure of kin17 are well known [19,20,21,22], its location and interaction with chromatin and/or the nuclear matrix in melanomas remains unclear.

Tumor cell lines and tumors growing *in vivo* are heterogeneous, and while some cells are only capable of invading adjacent tissues, other cells metastasize to distant locations. Limiting dilution was used to isolate some clones from the murine melanoma cell line B16F10-Nex2 that developed preferentially at the subcutaneous or metastatic sites [1]. Differences in the metastatic capacities of the clones could be related to the pattern of cathepsin release/accumulation [1], but other molecules were not evaluated.

The aim of this study was to evaluate the expression of the kin17 protein in the nuclear compartments (either associated or not associated to chromatin) of the B16F10-Nex2 melanoma cell line and derived clones with different metastatic capacities to identify whether the protein localization is correlated with the metastatic potential of these cells.

## 2. Results

### 2.1. Isolation of Low and High Metastatic Tumor Cells

**Figure 1:**
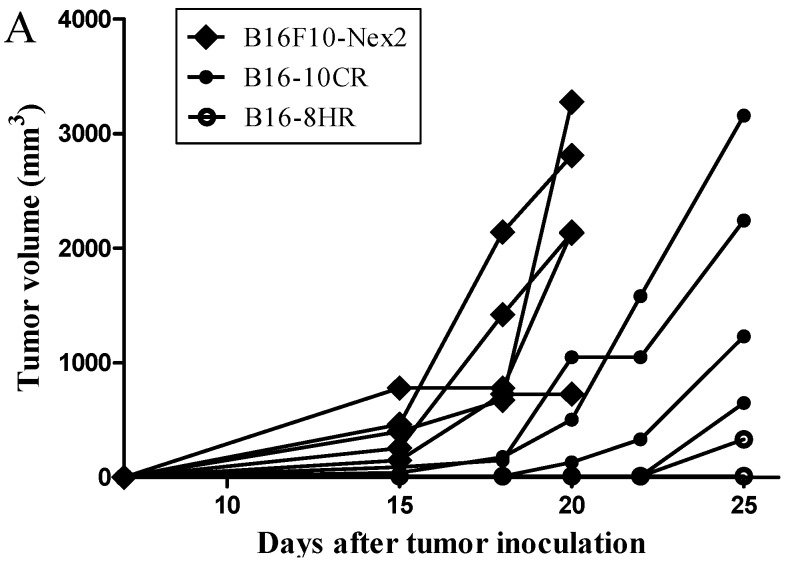

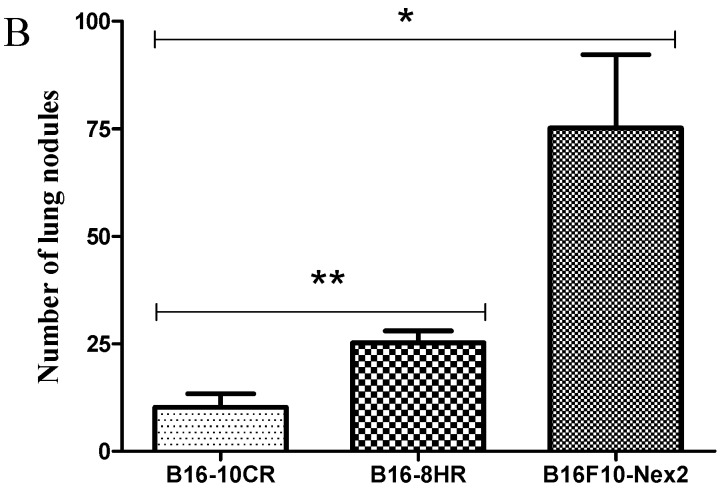
Clones with different metastatic properties were isolated from the B16F10-Nex2 murine melanoma cell line. (**A**) Local subcutaneous tumor development in syngeneic C57Bl/6 mice following injection with the B16F10-Nex2 cell line (*n* = 5) and clones B16-10CR (*n* = 4) and B16-8HR (*n* = 5), isolated by limiting dilution from B16F10-Nex2 cells. Tumor size is plotted separately for each animal; (**B**) The number of lung nodules in C57Bl/6 mice 15 days after intravenous inoculation with the B16F10-Nex2 cell line and clones B16-10CR and B16-8HR (*n* = 4 for all). The mean and standard deviation of each group is presented. * *p* = 0.0061; ** *p* = 0.0108.

As tumor cell lines are heterogeneous, clones with different properties can be isolated. Freitas *et al.* [1] isolated clones with different metastatic potential from the murine melanoma cell line B16F10-Nex2, and those clones expressed different levels of cathepsins. To determine whether kin17 protein expression in melanoma cells varies based on metastatic capacity, clones with different metastatic potential were isolated from the B16F10-Nex2 melanoma cell line by limiting dilution, following the established protocol described in [1]. After subcutaneous injection, B16F10-Nex2 cells developed local tumors in five of five syngeneic C57Bl/6 male mice, with tumors detected beginning on the seventh day after injection. Clone B16-10CR developed primary tumors in four of four syngeneic C57Bl/6 male mice, with tumor detection beginning on the 18th day after tumor injection, while clone B16-8HR developed subcutaneous tumors in only one of five injected animals, with the tumor detected only 25 days after tumor cell inoculation (Figure 1A). In contrast, after intravenous inoculation with B16F10-Nex2 cells, representing a metastatic model, clone B16-8HR produced significantly more lung nodules compared to clone B16-10CR (Figure 1B). These results show that clones B16-10CR and B16-8HR, isolated from the murine melanoma B16F10-Nex2 cells, exhibit low and high metastatic capacity, respectively.

### 2.2. Immunodetection of the kin17 Protein in Murine Tumor Cell Lines

**Figure 2:**
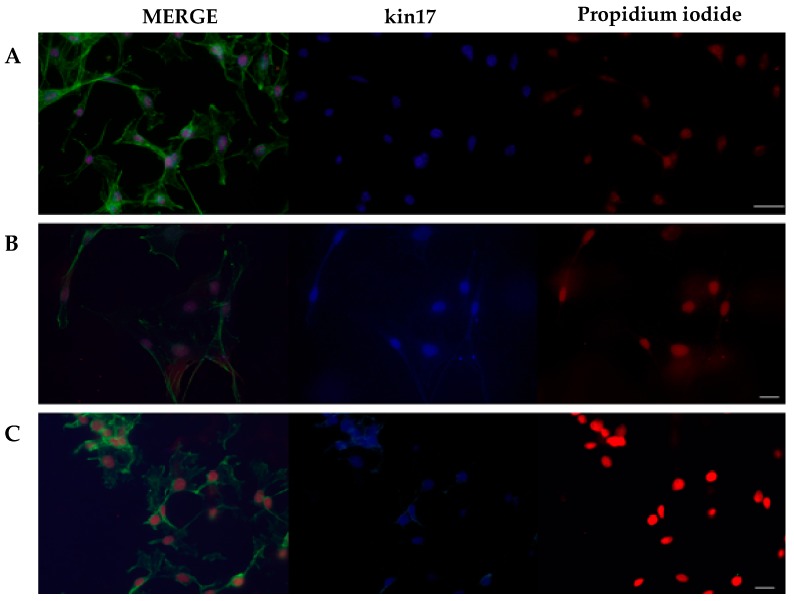
Immunodetection of the kin17 protein in murine melanoma tumor cells. B16F10-Nex2 cell line (antibody diluted 1:60), scale bar: 26µm (**A**); B16-8HR (1:40), scale bar: 16 µm (**B**) and B16-10CR (1:40), scale bar: 16 µm (**C**). Green, Alexa Fluor 488-conjugated Phalloidin; Red, propidium iodide; Blue, mouse anti-kin17 K58 clone followed by Alexa Fluor 350-conjugated goat anti-mouse.

The kin17 protein was immunolocalized in murine melanoma tumor cells. The cytoplasmic microtubules were detected by reaction with phalloidin (green), nuclei were localized by reaction with propidium iodide (red), and the kin17 protein was recognized by a monoclonal antibody (blue) (Figure 2A–C). Our analysis confirmed kin17 nuclear localization in B16F10-Nex2 cells and in the derived clones B16-8HR (high metastatic capacity) and B16-10CR (low metastatic capacity).

### 2.3. Detection of kin17 in Protein Extracts

**Figure 3:**
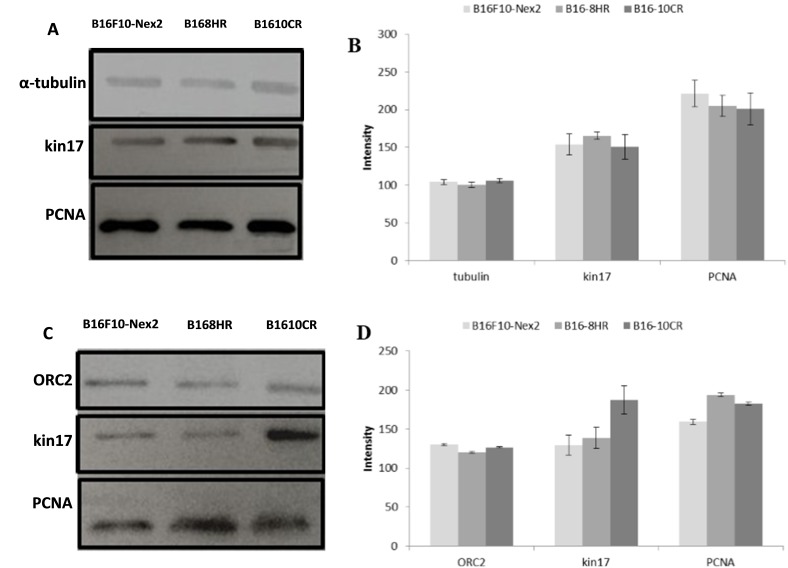
Kin17 protein detection by Western blotting in the nuclear compartments of the melanoma cell line B16F10-Nex2 clone B16-8HR and clone B16-10CR. (**A**) Expression of α-tubulin, kin17, and PCNA in nuclear protein fractions; (**B**) Densitometry measurements of α-tubulin, kin17, and PCNA proteins; (**C**) Expression of ORC2, PCNA, and kin17 in the chromatin-associated protein fractions; (**D**) Densitometry measurements of the ORC2, kin17, and PCNA proteins.

The melanoma cell line B16F10-Nex2 and its derived clones B16-8HR and B16-10CR were also analyzed for kin17 expression in different nuclear compartments by Western blotting. Cellular proteins were fractionated to obtain the nuclear and chromatin-associated fractions. Analysis of nuclear samples showed that the kin17 protein was detected in all samples, with slightly higher expression observed in B16-8HR. No differences were observed for α-tubulin expression, used as a loading control. PCNA protein expression was slightly increased in the B16F10-Nex2 cell line and in the B16-8HR clone (Figure 3A,B).

Kin17 was also detected in the chromatin-associated fraction of the three melanoma cell lines, with increased expression observed in the low-metastatic clone B16-10CR. All three cell lines expressed similar amounts of ORC2, which was used as a control for the attached chromatin protein fractions, and PCNA detection indicates that cell proliferation was taking place.

The B16-8HR and B16-10CR clones exhibited higher PCNA expression than B16F10-Nex2 cells (Figure 3C,D).

## 3. Discussion

Using the same limiting dilution protocol described by Freitas *et al.* [1], we isolated two clones from the murine melanoma B16F10-Nex2 cell line with different metastatic capacities. Clone B16-8HR exhibits a high metastatic capacity, inducing tumor development in the lungs of all animals after intravenous inoculation, while only one out of five animals showed local tumor growth after subcutaneous inoculation. Clone B16-10CR exhibited a low metastatic capacity, developing tumors in all animals at the subcutaneous site and producing low numbers of metastatic lung nodules. The isolation of these two clones corroborates the heterogeneous nature of the B16F10-Nex2 melanoma tumor, which is comprised of several populations of cells with different characteristics [1].

This study confirmed the expression of kin17 in association with chromatin in this melanoma model, supporting the participation of this protein in processes including DNA repair and replication as previously described for other tumor cells [7]. In clone B16-8HR, which exhibits high metastatic capacity, kin17 was detected in the nuclei in association with chromatin, with slightly higher nuclear expression compared to the other tumor cells analyzed here. The low-metastatic clone B16-10CR, which better develops tumors at the primary subcutaneous site, also exhibited kin17 protein expression in all cell compartments, with greater expression observed in the chromatin-associated fraction. The melanoma cell line B16F10-Nex2, the source of the B16-10CR and B16-8HR clones, exhibited a mixed phenotype. The expression of kin17 in the nuclear compartment was similar to that of the B16-10CR clone, while the expression in the chromatin-associated fraction was similar to that of the B16-8HR clone, corroborating the heterogeneity of tumor cell lines.

In a previous study, Despras and collaborators [5] showed a comparison among 16 human cell lines isolated from different tumor types. They observed a wide range of kin17 protein expression, and the metastatic melanoma cell line Mewo showed the lowest level, slightly lower than the human breast adenocarcinoma cell line MCF-7, a type of tumor with an overexpression of this protein [4]. Our results obtained with murine melanoma cell lines cannot be compared to the study of Despras *et al.* [5] because a primary human melanoma cell line was not analyzed.

The association of the kin17 protein with the DNA replication and repair mechanism is described elsewhere [5,7,13]. Micolli *et al.* [23] also detected the kin17 protein associated with chromatin and the nuclear matrix in HeLa cells, and the binding of this protein to other structure(s) may depend on the cell cycle phase. The authors also describe that chromatin-associated kin17 is overexpressed during the S phase [24,25].

The expression of the PCNA protein in the nuclear compartment and the chromatin-associated protein expression was similar among the three cell types tested here, suggesting equivalent proliferating properties. PCNA belongs to the family of DNA sliding clamps and has previously only been attributed nuclear functions in proliferating cells [26]. Some authors view this protein as a DNA polymerase accessory protein involved in repair synthesis [27]. The major role of this protein is to recruit and retain the replicative DNA polymerases at the sites of DNA synthesis during DNA replication. PCNA forms a homotrimeric ring encircling and freely sliding along the DNA helix. PCNA interacts with a large number of accessory proteins and acts as a protein recruitment platform to coordinate the multiple enzymatic activities required for DNA replication and repair and cell cycle control [25,28]. The PCNA protein can be associated with the cellular activity in cancer cells. Naryzhny and Lee [29] showed that the detection of PCNA in the cytoplasm is associated with glycolysis pathway proteins and cytoskeleton integrity, and this association may be involved in the regulation of oncogenesis.

The origin recognition complex (ORC) is a six-subunit complex that acts as the initiator (the protein that selects the sites for subsequent initiation of replication) at eukaryotic origins of replication, and it is involved in chromosome segregation [30,31,32,33]. In our experiments, all three cell lines expressed similar amounts of the ORC2 protein. This indicates that despite their metastatic capacity, these cell lines retain important functions of this protein, such as the prevention of pre-Replicative Complex assembly during the S-phase by inactivating the first step in its assembly, a checkpoint control mechanism, and another function related to replication previously described by DePamphilis [34].

The analysis of melanoma cell lines has become an excellent model for the identification of molecular changes associated with the metastatic phenotype as the disease progresses [35]. Analysis of gene patterns associated with the development of this disease may enable the development of a diagnostic manual to aid in disease identification and prognosis determination [2]. Many studies have aimed to identify molecular markers for melanoma diagnosis, with various miRNAs and HMGA proteins proposed as diagnostic markers that could enable faster, more efficient, and less invasive diagnosis [36,37]. The kin17 protein has been described as a potential diagnostic biomarker for breast cancer [4], which suggests that an analysis in human melanoma tissues is important to determine whether the level of kin17 could also be used as a biomarker for the identification of tumors with low and/or high metastatic capacity.

## 4. Experimental Section

### 4.1. Cell Lines and Culture Conditions

In this study, we used the murine melanoma cell line B16F10-Nex2 and two derived clones (B16-10CR and B16-8HR) isolated by limiting dilution at Laboratório de Imunobiologia do Câncer, Departamento de Microbiologia, Imunologia e Parasitologia, Escola Paulista de Medicina, Universidade Federal de São Paulo (EPM-UNIFESP), following the protocol described in [1]. The cells were cultured in RPMI-1640 medium, pH 7.2, supplemented with 10 mM HEPES, 24 mM sodium bicarbonate, 40 mg/mL gentamicin, and 10% fetal bovine serum. All cells were maintained at 37 °C and 5% CO_2_.

### 4.2. In Vivo Assays

B16F10-Nex2, B16-10CR, and B16-8HR cells were washed with PBS, diluted in RPMI medium without fetal bovine serum, and injected subcutaneously (5 × 10^4^) or intravenously in the tail vein (5 × 10^5^). Primary subcutaneous tumors were measured every three days with a calliper and tumor volumes were calculated using the formula: 0.52 × *d*^2^ × *D*, where *d* and *D* represent the short and long diameter, respectively. Lung metastatic melanotic nodules were counted 15 days after intravenous inoculation.

### 4.3. Immunodetection

B16F10-Nex2, B16-8HR, and B16-10CR cells were grown on coverslips for 6 to 8 h under the conditions described above. Cells were fixed with 4% paraformaldehyde for 10 min, permeabilized by Triton X-100 0.5% for 10 min, and blocked with PBS containing 3% BSA and 20% goat serum for one hour. The primary antibody anti-kin17 K58 (sc-32769; Santa Cruz Biotechnology, Dallas, TX, USA) was diluted 1:500 in blocking solution and then added to the cells for 1 h. Alexa Fluor 350-conjugated goat anti-mouse secondary antibody (A11045; Molecular Probes; Waltham, MA, USA) was diluted 1:4000 and incubated for one hour. To localize the cytoplasmic microtubules, cells were stained for 20 min with Alexa Fluor 488-conjugated Phalloidin (A12370; Molecular Probes) diluted 1:40, and nuclear chromatin was stained with propidium iodide (4 mg/mL) for 5 min. All staining reactions took place at 37 °C. Images were obtained using an Olympus FSX-100 microscope (Olympus, Tokyo, Japan).

### 4.4. Protein Extraction

The fractionation was performed as described by Méndez and Stillman [38]. The cell suspension was centrifuged at 600× *g* for 5 min, resuspended in 1× PBS, and centrifuged again at 600× *g* for 5 min. The cell pellet was recovered in buffer A (10 mM Hepes pH 7.9, 10 mM KCl, 21.5 mM MgCl, 0.34 M sucrose, 10% glycerol, 1 mM DTT, 0.1% Triton X-100, 1% protease inhibitor (Complete Protease inhibitor cocktail tablets, Roche, Mannheim, Germany)), incubated for 5 min at 4 °C, and then centrifuged for 4 min at 1300× *g.* In this protocol, the supernatant represents the cytoplasmic fraction.

The pellet was then recovered in buffer B (3 mM EDTA, 0.2 mM EGTA, 1 mM DTT, 1% protease inhibitor), incubated for 10 min at 4 °C, and centrifuged for 4 min at 1700× *g*. The nuclear protein fraction was collected in the supernatant. To the pellet, formed by the chromatin and its associated proteins, 150 μL of protein sample buffer (1:3 Red Loading Buffer, BioLabs, Ipswich, MA, USA) was added and the nucleic acids were digested with 1 μL of 250 U/µL Benzonase Nuclease (Sigma–Aldrich, St. Louis, MO, USA), yielding the chromatin-associated protein fraction.

### 4.5. Western Blotting

Protein samples were separated by electrophoresis in a discontinuous polyacrylamide-unidimensional gel in the presence of sodium dodecyl sulphate (SDS-PAGE), according to Laemmli [39]. A solution mix of acrylamide/bis-acrylamide (29:1) was used to prepare the stacking gel at a concentration of 5% (0.125 M Tris–HCl, pH 6.8, 0.1% SDS) and the separating gel at 10% (0.375 M Tris–HCl, pH 8.8; 0.1% SDS). Protein samples were incubated at 95 °C for 5 min in Red Loading Buffer, and the electrophoretic separation was performed at 100 volts for approximately 90 min. The proteins were then transferred to a nitrocellulose membrane, and efficiency was evaluated by Ponceau S staining, removed with TBS-T 1X (Tris–HCl 100 mM pH 7.5; NaCl 1.5 M; Tween-20 0.05%). Membranes were blocked using a 5% bovine albumin solution in 1× TBS-T for 60 min at 25 °C. Detection was performed using the anti-kin17 K58 antibody (sc-32769, Santa Cruz Biotechnology) or anti-kin17 K36 (sc-32768, Santa Cruz Biotechnology), diluted 1:15,000 in blocking solution, for 16 h at 4 °C, with similar results obtained for both antibodies. Anti-ORC2 (ab68348, Abcam, Cambridge, UK), anti-alpha-tubulin (ab52866, Abcam), and anti-PCNA (ab29, Abcam) antibodies were used at a 1:5000 dilution. The membranes were then washed in blocking solution and incubated with the secondary antibody (goat anti-mouse HRP Dako, P0447 or goat anti-rabbit HRP Dako, P0448 1:20,000 diluted) for one hour at room temperature. The membranes were stained using the ECL Prime kit (GE Healthcare, Little Chalfont, UK), and signals captured by the GE Image Quant LAS 500 system. Band quantification was performed by ImageJ (Wayne Hasband, National Institute of Health, Bethesda, MD, USA).

## 5. Conclusions

Our results indicate that the kin17 protein is present in the nucleus and associated with the chromatin in melanoma cell lines, reinforcing the involvement of this protein in processes such as transcription, DNA replication, and repair, as previously described. An increased concentration of the kin17 protein was observed in the chromatin-associated protein fraction of the low-metastatic cell clone B16-10CR.

Previous studies described kin17 as a potential diagnostic biomarker for breast cancer. The results shown here for melanoma cells suggest that the level of kin17 could also be used as a biomarker to identify tumors with low and/or high metastatic capacity. Further studies should be performed to confirm whether kin17 can be used as a melanoma diagnostic biomarker.
